# Endoscopic ultrasound-guided mucosal dissection to remove an entirely embedded esophageal fishbone

**DOI:** 10.1055/a-2530-3240

**Published:** 2025-03-25

**Authors:** Hongna Lu, Xiaolin Chen, Xiaofeng Feng, Feng Xu

**Affiliations:** 1 Department of Gastroenterology, The Affiliated LiHuili Hospital of Ningbo University, Ningbo, China; 2 Department of Gastroenterology, First Affiliated Hospital, Southwest Hospital, Army Medical University (Third Military Medical University), Chongqing, China

A 56-year-old woman presented with swallowing pain after eating fish. She underwent laryngoscopy at a local hospital, which did not reveal the fishbone. Consequently, she was referred to our hospital for further investigation.

**Fig. 1:**
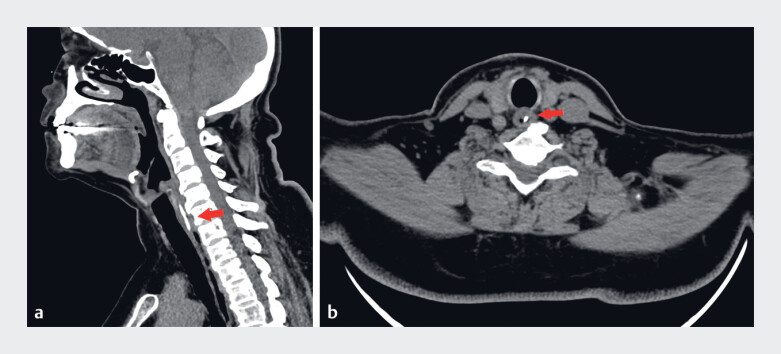
Preoperative computed tomography scan revealing the location of the fishbone (arrow). **a** Sagittal plane. **b** Transverse plane.

**Fig. 2:**
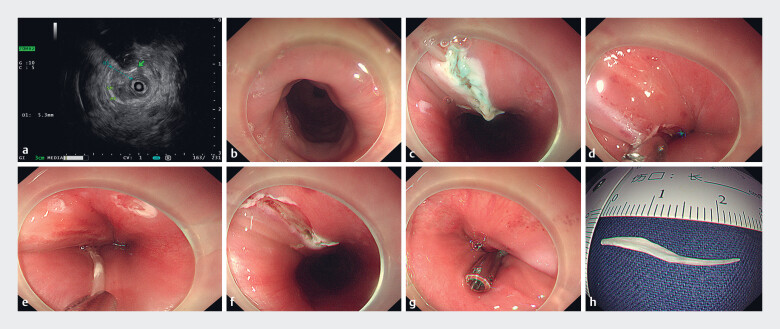
Endoscopic ultrasound (EUS)-guided mucosal dissection to remove an entirely embedded esophageal fishbone. **a** The foreign body was identified via EUS to be situated above the intrinsic muscular layer of the anterior wall of the esophageal inlet. **b** On gastroscopy, no foreign body was seen in the esophageal lumen. **c** The end of the foreign body was exposed after incision of the mucosal layer. **d** A thermal biopsy forceps was used to grasp the end of the foreign body. **e** The foreign body was removed. **f** The wound exhibited no notable perforation or hemorrhage. **g** The wound was closed using titanium clips. **h** The foreign body specimen.

A computed tomography (CT) scan of the neck revealed a 25-mm high-density foreign body at the entrance of the esophagus ( Fig. 1 ). On gastroscopy, no foreign body was observed within the bilateral pyriform fossa, esophageal inlet, or the entire lumen of the esophagus ( Fig. 2 **b** ). These findings suggested that the foreign body had become entirely embedded in the esophageal wall.

The foreign body was identified via endoscopic ultrasound (EUS) to be situated above the intrinsic muscular layer of the anterior wall of the esophageal inlet ( Fig. 2 **a** ). A disposable mucosal cutting knife was used to incise the mucosal layer at the site of the foreign body, thereby exposing the end of the foreign body ( Fig. 2 **c** ). Subsequently, the foreign body was extracted using a thermal biopsy forceps ( Fig. 2 **d, e** ). The wound exhibited no notable perforation or hemorrhage ( Fig. 2 **f** ). Finally, the wound was closed using titanium clips ( Fig. 2 **g** , Video 1 ).

The patient was administered postoperative symptomatic supportive treatment, including fasting and acid suppression, and was successfully discharged on the third postoperative day.

The primary challenge in the removal of a foreign body embedded in the esophageal wall is the accurate localization of the lesion and the precise determination of the incision site. The use of EUS facilitates the precise localization of the incision site and depth of incision, thereby minimizing the risk of secondary injury. The esophageal inlet represents one of the physiologic strictures of the esophagus, characterized by a narrow and confined space that presents a significant challenge for observation and endoscopic manipulation. Furthermore, water injection at the esophageal inlet during EUS carries an increased risk of reflux aspiration. The foreign body described in this article was entirely embedded in the wall of the esophageal inlet and was subsequently removed via EUS-guided mucosal dissection.

Endoscopic ultrasound-guided mucosal dissection to remove an entirely embedded esophageal fishbone.Video 1

Endoscopy_UCTN_Code_TTT_1AO_2AL

